# A dataset on habitat-associated changes in the fecal microbiota of *Drosophila melanogaster*

**DOI:** 10.1038/s41597-026-07673-7

**Published:** 2026-06-30

**Authors:** Elisabeth K. Riedel, Marko Rohlfs

**Affiliations:** Institute of Ecology, University of Bremen, Bremen, Germany

## Abstract

*Drosophila melanogaster* is a flagship model for studying animal-microbe interactions. In nature, its larvae develop on ephemeral plant substrates, where growth depends on the presence and metabolic activity of bacterial and fungal symbionts. Yet, microbial communities associated with natural breeding sites differ markedly from those maintained in laboratory cultures on artificial media. Most research on *Drosophila*-microbe interactions has been conducted under controlled but artificial conditions, limiting our understanding of the ecological and evolutionary dynamics of these associations. To bridge this gap, we re-associated laboratory fly cultures with field-exposed plant substrates, establishing semi-natural microcosms that harbor diverse, substrate-specific microbiota. Microcosms were sustained by fly-mediated transfer of microbial communities through fecal deposition onto new plant substrates. We present a comprehensive metabarcoding dataset of *D. melanogaster* fecal microbiota, including both bacterial (16S V3–V4) and fungal (ITS2) communities. To capture temporal dynamics, six plant substrate-based microcosms – apple, tomato, lemon, grape, onion, and plum – were sampled three times over five months, providing strain-level resolution of symbiont community composition.

## Background & Summary

Microbes profoundly influence host physiology, behavior, and reproduction, and their diversity and composition can determine the stability and outcomes of host-microbe interactions. Although *Drosophila melanogaster* is a cornerstone model for host-microbe research^1^, there is a scarcity of data sets bridging ecological realism with experimental tractability. Most studies use standardized rearing diets and simplified microbial communities^2–4^, underrepresenting the diversity and complexity found in the insects’ natural habitat^5^. This gap is particularly pronounced for fungal symbionts, despite their recognized nutritional and ecological importance^6,7^.

Here, we present a data set characterizing both bacterial and fungal communities associated with *D. melanogaster* from semi-natural microcosms, including variation across substrates, sampling years, and microbe transmission histories^8^. These data provide a resource for exploring microbial diversity, composition, and ecological variability in a key model organism, supporting research on the environmental drivers of host-microbe interactions^5,9–13^. Beyond the contribution of maternally inherited endosymbionts, bacteria and fungi are also introduced during oviposition via fecal-mediated microbial seeding, whereby adult flies deposit fecal material containing viable microbes onto the plant substrate^14,15^. These transmitted microbes establish in the substrate and profoundly alter the nutritional and chemical properties^16^, thereby influencing larval growth trajectories, developmental timing, and survival, with lasting effects on adult size, physiology, and overall fitness^17–21^.

For this data set^8^, we established microcosms to maintain *Drosophila*-associated microbial communities across multiple generations, using six different breeding substrates: apples, plums, grapes, lemons, tomatoes, and onions. These substrates were chosen because they are naturally colonized by *Drosophila* in the field^22,23^. *Drosophila melanogaster* developed on all of them, despite differences in sugar content and pH, which likely shape the microbes each substrate supports. In contrast, cucumber proved unsuitable as a breeding substrate^22,23^. To allow natural microbial colonization, the substrates were first exposed in the field and then transferred to the laboratory. Over five months, we sampled the fecal microbiota of female flies associated with these microbiota in the microcosms, replenishing substrates on a weekly basis. We chose three time points to reveal potential changes in microbial composition across fly generations. High-throughput sequencing of bacterial (16S rRNA amplicon) and fungal (ITS amplicon) communities provided a comprehensive view of microbial diversity and transmissibility.

**Fig. 1:**
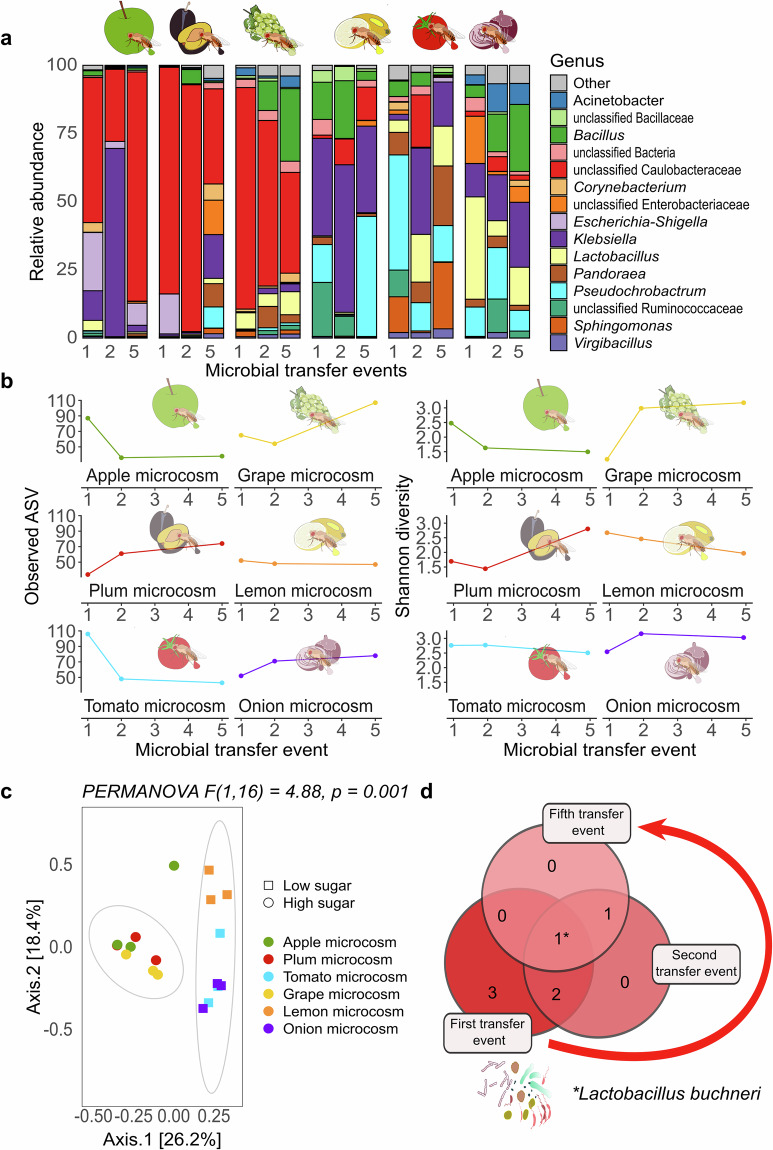
Bacterial microbiota of *Drosophila melanogaster* based on 16S rRNA gene amplicon sequencing (metabarcoding). Microcosms established from field-collected, fly associated microbes were sampled using fecal material of groups of 30 female flies maintained on a single substrate type (apple, plum, grape, lemon, tomato, onion). For each substrate, three samples were collected over time under laboratory conditions. (**a**) Stack plots showing the 15 most abundant bacterial genera in fecal samples. (**b**) Alpha diversity dynamics over time, including observed taxa and Shannon diversity (amplicon sequence variant level). (**c**) Community composition of the bacterial microbiota grouped by substrate sugar content (data from the USDA FoodData Central^31^); 95% confidence intervals are drawn as ellipses. (**d**) Venn-diagram of the core microbiota at a detection threshold of 0.001 (0.01% of reads) and a prevalence of 0.8 across samples.

**Fig. 2:**
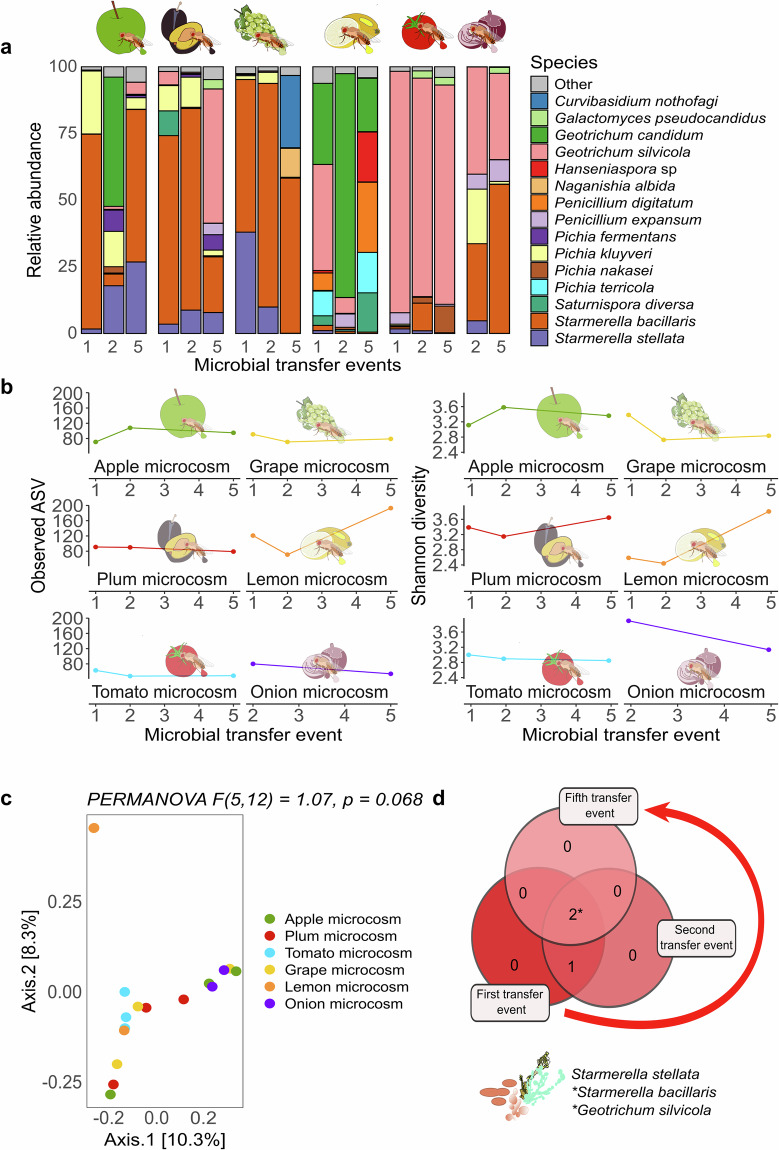
Fungal microbiota of *Drosophila melanogaster* based on ITS2 amplicon sequencing (metabarcoding). Microcosms established from field-collected, fly-associated microbes were sampled using fecal material from groups of 30 females maintained on a single substrate type (apple, plum, grape, lemon, tomato, onion). For each substrate, three samples were collected over time under laboratory conditions. (**a**) Stack plots showing the 15 most abundant fungal taxa in fecal samples. (**b**) Alpha diversity dynamics over time, including observed taxa and Shannon diversity (amplicon sequence variant level). (**c**) Community composition of fungal microbiota across substrate types. (**d**) Venn-diagram of the core microbiota at a detection threshold of 0.001 (0.01% of reads) and prevalence of 0.8 across samples.

The data show that *Drosophila*-associated microbial communities are diverse and dynamic, including at least 15 bacterial genera and 15 fungal species (Figs. 1a, 2a). Both fungal and bacterial species count, and Shannon diversity varied across substrate type during their maintenance under laboratory conditions (Figs. 1b, 2b). Sugar content appeared to be a primary factor influencing bacterial community structure, with substrates clustering into high- and low-sugar groups (Fig. 1c). Fungal communities were more conserved on some substrates than on others, however, the pattern was not strong enough to be statistically significant in a PERMANOVA test (Fig. 2c). Notably, *Lactobacillus buchneri* was the only bacterial strain consistently associated across all fly populations, independent of substrate or sampling time (Fig. 1d). *Geotrichum silvicola* and *Starmerella bacillaris* were the only fungal taxa conserved across all samples (Fig. 2d).

The initial fruit collection and resulting microcosms also included cucumber as a substrate. However, cucumber did not support the full life cycle of *D. melanogaster* as previously suggested by observations made by Atkinson & Shorrocks (1977)^22^. Early larval stages were occasionally observed, but development stopped after a few transfers, leading to population collapse. No fecal material could therefore be collected from this treatment for microbial community analysis. These findings indicate that cucumber is a poor or unsuitable substrate for sustaining *D. melanogaster* populations. Possible explanations include low nutrient availability, toxicity of the substrate, or the growth of microbes that produce harmful secondary metabolites. Another factor could be the failure to establish beneficial microbial consortia that not only suppress pathogens or detoxify harmful plant metabolites, but also provide essential nutrients for larval growth and development.

Part of the data has been analyzed for a manuscript in preparation. This includes fecal samples from flies reared in apple or tomato microcosms, three of which are shown here (Figs. 1, 2), as well as earlier collections. Moreover, substrate samples were taken from single-larva environments after successful development in an experimental setup described in Riedel *et al*.^24^. The aim was to compare microbiota assembly from the initial inoculum to post-eclosion community across different substrates. For this purpose, single axenic larvae were placed in 2 mL collection tubes filled with apple, cucumber or tomato substrate inoculated with fecal material from flies collected from a tomato-based microcosm first established in September 2019. A parallel fecal sample was also collected and included in the data set. In this design, larvae developed either on a new substrate with an “allochthonous” microbial community (apple or cucumber) or on the original substrate with its “autochthonous” microbial community (tomato). In total, the data set includes 40 samples, 20 of which are described here, incl. two blanks (Figs. 1, 2).

## Methods

### Microcosm establishment

**Fig. 3:**
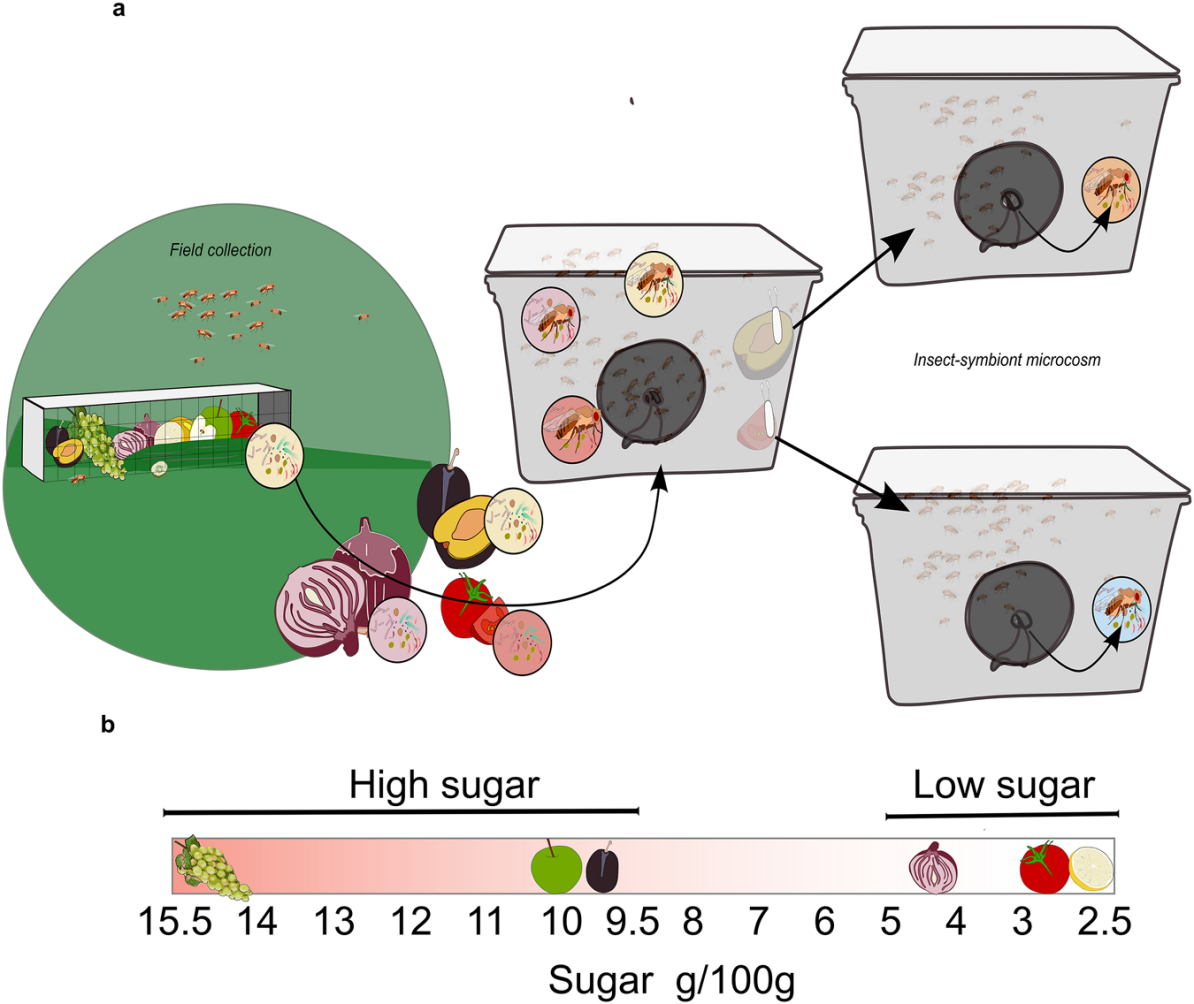
Microcosm establishment and substrate properties. (**a**) Schematic illustration of collection of microbes in the field and subsequent establishment of substrate-based microcosms in the laboratory. During field collection (left) different substrates including apple, plum, grape, tomato, onion and lemon were exposed to insects. After a week of exposure these substrates were offered to a laboratory-reared, outbred *D. melanogaster* population, so flies would take up some of the microbiota (middle). In the same cage fresh substrates were offered as egg and microbiota disposition site. These substrates were then separated after type to different cages and flies were left to develop in the respective microbial community (right). (**b**) The six substrates used as substrate for the fly-microbe interaction differ in sugar content and can be grouped in fruits with high and low sugar content. Values were obtained from the USDA FoodData Central^31^.

In September 2020, microcosms were established to investigate microbial transfer in *Drosophila melanogaster* in different plant substrate environments. Substrate pieces (apple, plum, grape, onion, tomato, cucumber, and lemon) were enclosed in cages and used as bait. The cages were built from steel bookshelves with steel mesh on the long sides (98.5 cm × 20 cm × 26 cm, mesh size 0.5 cm × 0.5 cm). This design allowed visits from small to medium sized insects while protecting the baits from rain and larger animals e.g. mice and birds. Each bait substrate was placed on 10 cm-high plastic pedestals coated with Tangle-Trap paste (Temmen Insekten Leim) to exclude access by non-flying arthropods e.g. Collembola or woodlice. The baits were exposed for one week under sunny weather conditions in the “Biogarten” facilities of the University of Bremen, Germany (ca. 53°06′43.5″N, 8°51′08.5″E, Fig. 3a).

Following the exposure period, each substrate was manually inspected, and all arthropods were removed. Then substrates were placed in a separate cage containing a young – one to five days post-eclosion – outbred *D. melanogaster* population^25^. Endosymbiont infection status of this fly strain is currently undetermined. This allowed flies to interact with the substrates and acquire microbes over a 24-hour period. The old substrates were then replaced with fresh ones of the same type, allowing the flies to lay eggs and transfer the microbes they had picked up from the field substrates. These substrates were transferred to new cages to establish a new fly generation within each specific substrate-microbe environment. The substrates were replenished weekly to bi-weekly with up to 150 g of chunky-cut (halved or quartered) fruit or vegetable pieces to ensure unlimited availability for the developing larvae (Fig. 3a, insect-symbiont microcosms). All substrates were purchased from a local organic supermarket (ALECO BioMarkt HB-Universität, Bremen, Germany).

Microcosms were maintained in cages under our standard rearing conditions for *D. melanogaster*: 16:8 light-dark cycle at 20 ± 2 °C at a density of several hundred adult individuals. Adult flies in the microcosms were additionally provided with water and a yeast-sugar mixture to sustain egg production. Approximately once per month, new cages were set up, marking a single microbiota transfer event. In this way, the microbiota and associated organisms were carried through successive generations, depending on the successful emergence of adult flies in each microcosm. The cucumber microcosms never produced enough flies to yield fecal material for analysis, confirming this substrate as unsuitable for sustaining *D. melanogaster* across generations.

In December of 2020, free-living nematodes were detected in the microcosms and later identified as *Panagrellus redivivoides* (GenBank accession numbers PV054487 and PV054488).

### Collection of fecal samples from female *Drosophila melanogaster*

Fecal material of female flies is an identified route of intergenerational transfer of microbes that enrich the fresh fruit and vegetables to be ontogenetic environments for juvenile flies that sustains their development^5,14,15^. Fecal samples from female *D. melanogaster* were collected after 1, 2, and 5 microbiota transfer events (three samples from each of the six microcosms, n = 18). For this, thirty females were removed from each microcosm using an indirect suction method with a vacuum cleaner. The flies were transferred to15 mL screw-cap tubes and kept overnight, during which they deposited fecal droplets that provided microbiota samples^5^. For metabarcoding analysis, the tubes containing fecal samples were immediately frozen at −80 °C and stored at this temperature until DNA extraction.

We included four additional fecal samples from conventionally reared flies, divided into two groups. The first group consisted of two samples taken from flies maintained on a standard cornstarch agar diet (12 g agar, 50 g refined sugar, 10 mL methyl-4-hydroxybenzoate [10% w/v in ethanol], 10 mL sorbic acid [10% w/v in ethanol], 250 g apple purée, 50 g cornmeal, 70 g brewer’s yeast in 600 mL water). The second group consisted of two samples from flies transferred to thawed organic raspberries, as described in Cho and Rohlfs^14^. These flies were allowed to associate with the fruit for 24 hours before fecal samples were collected. Together, these samples provide a reference for microbes originally harbored by the laboratory culture and their potential to develop on fruits without prior exposure to field microbiota.

The remaining samples in this data set^8^ are described in Riedel *et al*.^24^. In short, microcosms created in 2019, including one based on banana as one substrate type, were sampled as described above. We took two fecal samples from apple and banana microcosms, and three fecal samples from tomato microcosms. In addition, substrate samples were collected from experiments inoculated with one of these fecal samples and then sampled again after successful fly development^24^. An overview over the samples is given in Tables 1, 2.

**Table 1:** Samples accession numbers and descriptions.

Sample accession	Sample ID	Library	Cage of origin	Substrate type	Collection year	Sample type
SAMEA120098820	1	16S	Apple-2019	Apple	2019	fecal
SAMEA120103216	1	ITS	Apple-2019	Apple	2019	fecal
SAMEA120098819	2	16S	Banana-2019	Banana	2019	fecal
SAMEA120103215	2	ITS	Banana-2019	Banana	2019	fecal
SAMEA120098818	3	16S	Tomato-2019	Tomato	2019	fecal
SAMEA120103214	3	ITS	Tomato-2019	Tomato	2019	fecal
SAMEA120098817	4	16S	Apple-2019	Apple	2019	fecal
SAMEA120103213	4	ITS	Apple-2019	Apple	2019	fecal
SAMEA120098816	5	16S	Banana-2019	Banana	2019	fecal
SAMEA120103212	5	ITS	Banana-2019	Banana	2019	fecal
SAMEA120098815	6	16S	Tomato-2019	Tomato	2019	fecal
SAMEA120103211	6	ITS	Tomato-2019	Tomato	2019	fecal
SAMEA120098814	7	16S	Apple-2020	Apple	2020	fecal
SAMEA120103210	7	ITS	Apple-2020	Apple	2020	fecal
SAMEA120098813	8	16S	Plum-2020	Plum	2020	fecal
SAMEA120103209	8	ITS	Plum-2020	Plum	2020	fecal
SAMEA120098812	9	16S	Tomato-2020	Tomato	2020	fecal
SAMEA120103208	9	ITS	Tomato-2020	Tomato	2020	fecal
SAMEA120098811	10	16S	Grape-2020	Grape	2020	fecal
SAMEA120103207	10	ITS	Grape-2020	Grape	2020	fecal
SAMEA120098810	11	16S	Lemon-2020	Lemon	2020	fecal
SAMEA120103206	11	ITS	Lemon-2020	Lemon	2020	fecal
SAMEA120098809	12	16S	Onion-2020	Onion	2020	fecal
SAMEA120103205	12	ITS	Onion-2020	Onion	2020	fecal
SAMEA120098808	13	16S	Apple-2020	Apple	2020	fecal
SAMEA120103204	13	ITS	Apple-2020	Apple	2020	fecal
SAMEA120098807	14	16S	Plum-2020	Plum	2020	fecal
SAMEA120103203	14	ITS	Plum-2020	Plum	2020	fecal
SAMEA120098806	15	16S	Tomato-2020	Tomato	2020	fecal
SAMEA120103202	15	ITS	Tomato-2020	Tomato	2020	fecal
SAMEA120098805	16	16S	Grape-2020	Grape	2020	fecal
SAMEA120103201	16	ITS	Grape-2020	Grape	2020	fecal
SAMEA120098804	17	16S	Lemon-2020	Lemon	2020	fecal
SAMEA120103200	17	ITS	Lemon-2020	Lemon	2020	fecal
SAMEA120098803	18	16S	Onion-2020	Onion	2020	fecal
SAMEA120103199	18	ITS	Onion-2020	Onion	2020	fecal
SAMEA120098802	19	16S	Apple-2020	Apple	2020	fecal
SAMEA120103198	19	ITS	Apple-2020	Apple	2020	fecal
SAMEA120098801	20	16S	Plum-2020	Plum	2020	fecal
SAMEA120103197	20	ITS	Plum-2020	Plum	2020	fecal
SAMEA120098800	21	16S	Tomato-2020	Tomato	2020	fecal
SAMEA120103196	21	ITS	Tomato-2020	Tomato	2020	fecal
SAMEA120098799	22	16S	Grape-2020	Grape	2020	fecal
SAMEA120103195	22	ITS	Grape-2020	Grape	2020	fecal
SAMEA120098798	23	16S	Lemon-2020	Lemon	2020	fecal
SAMEA120103194	23	ITS	Lemon-2020	Lemon	2020	fecal
SAMEA120098797	24	16S	Onion-2020	Onion	2020	fecal
SAMEA120103193	24	ITS	Onion-2020	Onion	2020	fecal
SAMEA120098796	25	16S	Lab-diet	Cornstarch-medium	2021	fecal
SAMEA120103192	25	ITS	Lab-diet	Cornstarch-medium	2021	fecal
SAMEA120098795	26	16S	Lab-diet	Cornstarch-medium	2021	fecal
SAMEA120103191	26	ITS	Lab-diet	Cornstarch-medium	2021	fecal
SAMEA120098794	27	16S	Raspberry	Raspberry	2021	fecal
SAMEA120103190	27	ITS	Raspberry	Raspberry	2021	fecal
SAMEA120098793	28	16S	Raspberry	Raspberry	2021	fecal
SAMEA120103189	28	ITS	Raspberry	Raspberry	2021	fecal
SAMEA120098792	29	16S	Blank		2021	blank
SAMEA120103188	29	ITS	Blank		2021	blank
SAMEA120098791	30	16S	Blank		2021	blank
SAMEA120103187	30	ITS	Blank		2021	blank
SAMEA120098790	31	16S	Tomato-2019	Tomato	2020	fecal
SAMEA120103186	31	ITS	Tomato-2019	Tomato	2020	fecal
SAMEA120098789	32	16S	Cucumber post eclosion	Cucumber	2021	substrate
SAMEA120103185	32	ITS	Cucumber post eclosion	Cucumber	2021	substrate
SAMEA120098788	33	16S	Cucumber post eclosion	Cucumber	2021	substrate
SAMEA120103184	33	ITS	Cucumber post eclosion	Cucumber	2021	substrate
SAMEA120098787	34	16S	Cucumber post eclosion	Cucumber	2021	substrate
SAMEA120103183	34	ITS	Cucumber post eclosion	Cucumber	2021	substrate
SAMEA120098786	35	16S	Apple post eclosion	Apple	2021	substrate
SAMEA120103182	35	ITS	Apple post eclosion	Apple	2021	substrate
SAMEA120098785	36	16S	Apple post eclosion	Apple	2021	substrate
SAMEA120103181	36	ITS	Apple post eclosion	Apple	2021	substrate
SAMEA120098784	37	16S	Apple post eclosion	Apple	2021	substrate
SAMEA120103180	37	ITS	Apple post eclosion	Apple	2021	substrate
SAMEA120098783	38	16S	Tomato post eclosion	Tomato	2021	substrate
SAMEA120103179	38	ITS	Tomato post eclosion	Tomato	2021	substrate
SAMEA120098782	39	16S	Tomato post eclosion	Tomato	2021	substrate
SAMEA120103178	39	ITS	Tomato post eclosion	Tomato	2021	substrate
SAMEA120098781	40	16S	Tomato post eclosion	Tomato	2021	substrate
SAMEA120103177	40	ITS	Tomato post eclosion	Tomato	2021	substrate

**Table 2:** Overview of samples included in the data set.

Cage of origin	Sample_no*	Substrate type	Collection year	Sample type	Samples over time	Aim
Apple–2020	7,13,19	Apple	2020	fecal	3	Microbiota after 1,2 and 5 transfer events
Plum–2020	8,14,20	Plum	2020	fecal	3	
Grape–2020	10,16,22	Grape	2020	fecal	3	
Lemon–2020	11,17,23	Lemon	2020	fecal	3	
Tomato–2020	9,15,21	Tomato	2020	fecal	3	
Onion–2020	12,18,24	Onion	2020	fecal	3	
Apple-2019	1,4	Apple	2019	fecal	2	Riedel *et al*.^24^
Tomato-2019	3,6	Tomato	2019	fecal	2	Riedel *et al*.^24^
Banana-2019	2,5	Banana	2019	fecal	2	Microbiota sample
Tomato-2019	31	Tomato	2019	fecal	1	Inoculate for substrate samples
Apple post eclosion	35,36,37	Apple	2021	substrate	3	Substrate samples after alloch- or autochthonous inoculation
Tomato post eclosion	38,39,40	Tomato	2021	substrate	3	
Cucumber post eclosion	32,33,34	Cucumber	2021	substrate	3	
Lab-diet	25,26	Cornstarch-medium	2021	fecal	2	Microbiota of laboratory fly culture
Raspberry	27,28	Raspberry	2021	fecal	2	Microbiota of laboratory culture after 24 h of association with raspberry substrate
Blank	29,30		2021	blank	2	Blank samples handled alongside fecal sample
Total					40	

*Sample_no as listed in PRJEB96953 and PRJEB96936 European Nucleotide Archive (ENA) at EMBL-EBI.

### DNA extraction and metabarcoding

DNA was extracted using the Qiagen DNeasy PowerSoil Kit (12888-100), following the method described in Fink *et al*.^26^. Briefly, the screw-cap tubes used for fecal matter collection were rigorously sampled using a sterile applicator (Boettger 09-119-0100) dipped in autoclaved phosphate buffered saline (1 liter of H_2_O dd, 8 g NaCl, 200 mg KCl, 1.44 g Na_2_HPO_4_, 245 mg KH_2_PO_4_, pH = 7.2 ± 0.2). The cotton tip was clipped into a Qiagen reaction tube using scissors sterilized with 70% ethanol.

To control for contamination, two empty screw-cap tubes handled alongside the fecal samples were included as blanks. Six of the samples, three from the tomato-based microcosm and three from the apple-based microcosm, are also reported in Riedel *et al*.^24^.

A total of 40 DNA samples were sent to Advanced Identification Methods (AIM) GmbH in Leipzig, Germany, for sequencing. Each sample was split for parallel fungal and bacterial dual-tag amplicon sequencing on an Illumina MiSeq v3.0 platform. For fungal species identification, the internal transcribed spacer 2 (ITS2) region was amplified using the ITS3_KYO2 and ITS4_KYO3 primer pair^27^. For bacterial identification, the variable regions V3-V4 of the 16S ribosomal RNA gene were amplified with the 341 F and 785 R primer pair^28^. Fungal and bacterial libraries were sequenced in two separate paired-end approaches (2x 300 bp). Dual tags were removed from the final sequences by the sequencing provider.

### Bioinformatic procedures for descriptions of *Drosophila melanogaster* microbiota

Demultiplexed sequences (included in the data set^8^) were separated according to the amplicon (fungal ITS or bacterial 16S), for each sample. Each batch was processed separately in QIIME 2 version 2022.2^29^ on a Linux virtual machine (Linux 5.4.0-148-generic), following the procedure described below.

All reads were denoised and paired using DADA2^30^. 16S amplicon reads were truncated at 299 (forward) and 255 bp (reverse). For the ITS amplicon, forward reads were not truncated, while reverse reads were truncated at 215 bp. Forward and reverse reads were paired, and primers were removed in the same step. Quality scoring during pairing was set to 2.0, except for fungal reverse reads where it was 5.0. 16S amplicon reads were additionally filtered for chimeras. Taxonomic classification was performed using naïve Bayes classifiers trained on the UNITE database (version 16.10.2022; dynamic taxonomy, 97–99% identity) and on a primer pair-based subset of the SILVA database (release 132; 99% identity). No steps were taken to exclude endosymbionts. After classification, the quality-filtered and paired sequences were exported from the QIIME2 environment.

A total of 133 unidentified Amplicon sequence variants (ASVs) from ITS reads were excluded after producing non-fungal hits in NCBI BLAST (BLAST + 2.13.0, December 2022) against the *Nucleotide collection (nr/nt)* database. Three fungal ASVs were identified and added to the data set: one Saccharomycetaceae species (one read, NCBI accession number: 7ZW0_LD, 100% coverage) and two *Penicillium chrysogenum* complex features (two reads each, accession number: MW774585.1, 98.39% coverage).

**Fig. 4:**
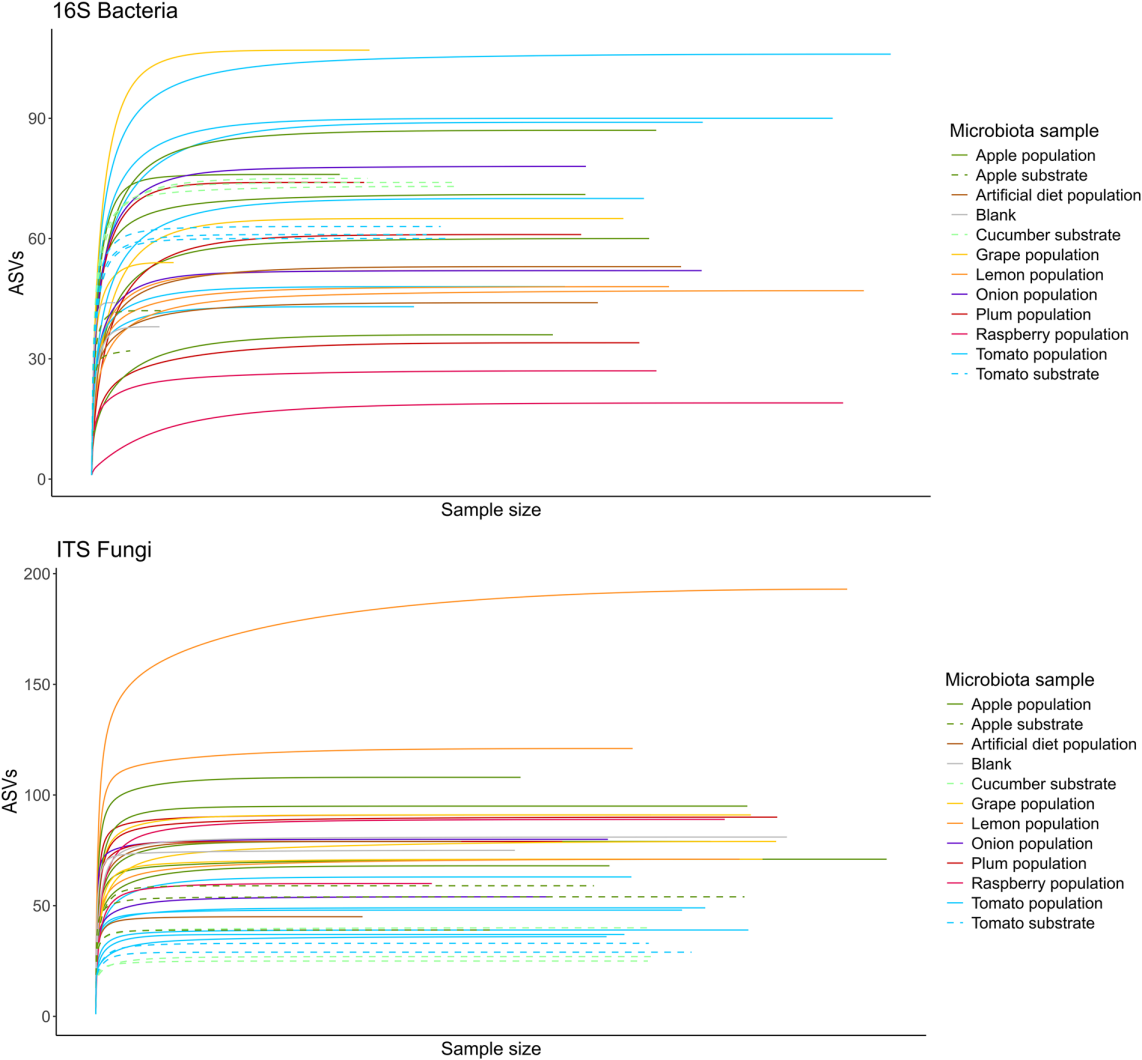
Rarefaction curves for 16S and ITS sequencing data of all forty samples. Rarefaction curves are shown for all samples, with the 16S dataset (top) and ITS dataset (bottom). Solid lines represent fecal samples from fly populations reared in microcosms containing a single substrate type (e.g., Apple population). Grey solid lines indicate blank control samples processed alongside fecal samples. Dashed lines represent substrate samples that were inoculated with fecal material and collected after fly development (e.g., Apple substrate).

All data were imported into the R environment (R Core Team, 2022; RStudio Team, 2020). Contaminants were identified for both amplicons using the *decontam* package, based on prevalence in the blank samples, and were excluded. Rarefaction curves (rarecurve, *vegan* package) were visually inspected for adequate sampling depth. All samples plateaued except one ITS sample (from the onion-based microcosm, first fecal transfer event), which was excluded from the analysis (Fig. 4). Singletons and non-bacterial sequences were removed from the 16S data set.

### Microbial community description

Relative abundance of the 15 most common bacterial genera (16S amplicon) and 15 most common fungal species (ITS amplicon) were plotted using plot_bar() function from the *phyloseq package* (Figs. 1a, 2a). Species richness (Shannon and observed indices) was calculated with the estimate_richness() (*phyloseq*) (Figs. 1b, 2b). Community composition (beta diversity) is analyzed with Bray-Curtis distance (*phyloseq*) and multidimensional scaling *vegan* package. PERMANOVA tests were run with adonis2() (*vegan)* (Figs. 1c, 2c). Sugar content of substrates was estimated from United States Department of Agriculture FoodData Central entries (ID: 577849, 362759, 1103512, 1102665, 2709168, 1103364, 1102693, 1103276)^31^. Sugar content was considered high between 9.1–15.48 g/100 g and low between 1.9–4.24 g/100 g (Fig. 3b). Core microbiota (Figs. 1d, 2d) were defined as those present in at least 80% of samples and at a relative abundance ≥ 0.1% per sample, using the function core_members() from the *microbiome* package. The bacterial libraries varied in their sequencing depth. To ensure that the prevalence of core taxa reflects biological patterns rather than differences in sequencing depth across samples, we rarefied to 9,400 reads (the sample minimum) using the ‘rarefy_even_depth()’ function in the *phyloseq* package.

## Data Records

The data from this study are available from reference eight^8^ (Umbrella project), in the European Nucleotide Archive (ENA) at EMBL-EBI under https://identifiers.org/ena.embl:PRJEB96953 (2025, ITS amplicon reads) and under https://identifiers.org/ena.embl:PRJEB96936 (2025, 16S amplicon reads). Six of the samples described here were also included in Riedel *et al*.^24^. That publication reports six of the nine substrate samples and two fecal samples each from apple- and tomato-based microcosms established in 2019. Table 1 lists all sample accession numbers and the available libraries.

## Data Overview

Table 2 gives an overview of all samples, including samples highlighted in Figs. 1, 2, as well as any additional samples in the data set. Samples are labeled with the respective sample number which can be cross referenced with Table 1 and naturally the data provided through ENA^8^. We also provide information on the year of microcosm establishment and the fruit or vegetable provided as substrate basis of the microcosm. The general aim in generating each of the samples is also declared. In Fig. 4 we show rarefaction curves of all samples after processing them as described in the methods section.

## Technical Validation

Two blank samples, handled alongside the fecal samples are included in the data set^8^. We used them to filter potential contaminants from the data set using the *decontam* package with the prevalence method.

## Usage Notes

This data set^8^ offers insights into the ecological context of *D. melanogaster-*microbe interactions and may have implications for studies on microbial inheritance, host-microbiome co-evolution, and the evolution of niche breadth.

Future work can use this data set to investigate microbial functions, microbe-microbe interactions, and potential applications in insect microbiome management. The data can also serve as a foundation for comparative studies across different *Drosophila* species or geographic regions, helping to advance our understanding of host-microbiome dynamics in wild populations. As reference databases such as SILVA and UNITE continue to expand, the data set^8^ can be reanalyzed to improve taxonomic resolution and precision.

## Data Availability

The data from this study are available in the European Nucleotide Archive (ENA) at EMBL-EBI under https://identifiers.org/ena.embl:PRJEB96953 (2025) and under https://identifiers.org/ena.embl:PRJEB96936 (2025).

## Code availability

No custom code was used in this study. All bioinformatic steps were performed using default settings if not stated otherwise in the methods. All figures were created in R studio using ggplot2 and optimized using Inkscape graphical software.
